# Machine learning analysis of the association between psychosocial risks and burnout syndrome in mining workers

**DOI:** 10.3389/fpubh.2026.1871339

**Published:** 2026-07-10

**Authors:** Juan Kenedy Ramirez, Mayron Antonio Candia-Puma, Mayra Alexandra Arratia-Corrales, Yannina Melissa Peña-Pinto, Angélica Corzo Salas de Valdivia, Roxana Jacqueline Candelaria Gutiérrez-Araníbar, José Antonio Llahuilla-Quea, Isabel García-Torres, Roberth Chuquimbalqui-Maslucán, Rommel Luis López-Alvarado, Edgar Robert Tapia-Manrique, Ricardo Angel Yuli-Posadas

**Affiliations:** 1 Escuela de Postgrado, Universidad Católica de Santa María, Arequipa, Peru; 2 Facultad de Enfermería, Universidad Católica de Santa María, Arequipa, Peru; 3 Facultad de Ciencias Farmacéuticas, Bioquímicas y Biotecnológicas, Universidad Católica de Santa María, Arequipa, Peru; 4 Universidad Nacional Mayor de San Marcos, Lima, Peru; 5 Universidad Nacional Daniel Alcides Carrión, Pasco, Peru

**Keywords:** artificial intelligence, mining, occupational health, psychosocial risks, work-related stress

## Abstract

**Introduction:**

Burnout syndrome represents a critical issue in occupational health, particularly in high-demand contexts such as mining, where physical, environmental, and psychosocial risks converge and affect workers' wellbeing and job performance. In this context, the study objective is to analyze the association between psychosocial risk factors and burnout syndrome among mining workers in Moquegua, Peru.

**Methods:**

A quantitative, analytical cross-sectional study with a non-experimental design was conducted. The study population consisted of 65 workers from a mining unit in Moquegua, Peru. Given the complete accessibility of the target population, a census approach was adopted, and all eligible workers were included in the study (*N* = 65). Validated instruments were used, including the SUSESO/ISTAS21 questionnaire for psychosocial risks and the Maslach Burnout Inventory. Data analysis involved descriptive and correlational statistics, multiple linear regression, and machine learning techniques.

**Results:**

The findings revealed significant associations between psychosocial factors particularly social support, leadership, and work–family conflict (double presence) and burnout. All analyzed factors demonstrated significant associations capacity, with double presence emerging as the most influential predictor. Furthermore, machine learning analyses identified relevant burnout-related patterns within the analyzed dataset, highlighting their effectiveness in identifying burnout-related patterns.

**Discussion:**

Burnout in mining is a multifactorial phenomenon influenced by organizational and psychosocial conditions. The results support the use of machine learning as an useful tool for identifying psychosocial risk patterns that may support prevention strategies, contributing to improved occupational health strategies in high-risk industrial settings.

## 1 Introduction

Burnout syndrome constitutes a critical issue in the field of occupational health, particularly in high-demand work environments such as mining, where workers are exposed simultaneously to physical, environmental, and psychosocial risks (1–3). This syndrome, characterized by emotional exhaustion, depersonalization, and reduced personal accomplishment, has been recognized as a chronic response to prolonged occupational stress, with adverse effects on mental health, job performance, and workplace safety (4–6). In complex industrial settings, factors such as exposure to contaminants, extended working hours, and adverse labor conditions significantly increase vulnerability to burnout, thereby requiring advanced analytical approaches to understand and anticipate its occurrence through innovative tools such as machine learning.

Burnout syndrome has been extensively studied through established theoretical frameworks that explain its development as a response to chronic occupational stress. Maslach and Jackson (7) conceptualized burnout as a multidimensional construct composed of emotional exhaustion, depersonalization, and reduced personal accomplishment, dimensions that continue to serve as the foundation for contemporary research. Subsequently, Leiter and Maslach (8) argued that burnout emerges when there is a persistent mismatch between workers and their work environment, particularly in areas such as workload, control, reward, community, fairness, and values. These contributions have been fundamental in understanding burnout not merely as an individual condition but as an organizational phenomenon influenced by work-related factors.

Several instruments have been developed to assess burnout syndrome in occupational settings. Among the most widely used are the Copenhagen Burnout Inventory (CBI), the Oldenburg Burnout Inventory (OLBI), and the Maslach Burnout Inventory (MBI). While the CBI focuses primarily on personal, work-related, and client-related exhaustion, and the OLBI evaluates exhaustion and disengagement, the MBI remains the most extensively validated and internationally recognized instrument for measuring burnout. Its multidimensional structure, encompassing emotional exhaustion, depersonalization, and personal accomplishment, has been consistently supported across diverse occupational contexts, making it the reference standard in burnout research (7, 9). This multidimensional approach is particularly relevant in occupational health studies because it enables a comprehensive assessment of the psychological consequences of chronic work-related stress.

Similarly, psychosocial risks have been evaluated through several instruments, including the Job Content Questionnaire (JCQ) developed by Karasek, the Effort–Reward Imbalance Questionnaire (ERI) proposed by Siegrist, and the Copenhagen Psychosocial Questionnaire (COPSOQ), which has become one of the most comprehensive tools for assessing psychosocial working conditions (10–12). Among these, the COPSOQ has gained broad international acceptance because it provides a multidimensional assessment of psychosocial work environments, integrating factors related to job demands, organizational resources, social relationships, leadership, and work–family interactions (12). This comprehensive perspective facilitates the evaluation of both risk and protective factors within organizations, allowing a more holistic understanding of workers' psychosocial experiences. The SUSESO/ISTAS21 questionnaire, derived from the COPSOQ framework and adapted for Spanish-speaking occupational contexts, has been widely implemented in Latin America, demonstrating satisfactory validity and reliability indicators across diverse occupational sectors (13). Consequently, its use provides a contextually appropriate and psychometrically sound approach for assessing psychosocial risks among mining workers.

The selection of the SUSESO/ISTAS21 and the Maslach Burnout Inventory for the present study was based on their strong theoretical foundations, extensive use in occupational health research, and demonstrated psychometric robustness (7, 13). The SUSESO/ISTAS21 enables the assessment of psychosocial dimensions closely aligned with the Job Demands–Resources framework, including psychological demands, active work and skill development, social support and leadership quality, compensation, and double presence (14, 15). These dimensions capture both occupational demands and organizational resources, which are considered fundamental determinants of employee wellbeing and burnout. Likewise, the MBI provides a comprehensive evaluation of burnout through its three core dimensions: emotional exhaustion, depersonalization, and reduced personal accomplishment (7, 9). Therefore, the combined use of these instruments facilitates an integrated analysis of psychosocial work conditions and burnout syndrome, which is particularly relevant in high-risk occupational environments such as mining, where workers are exposed simultaneously to demanding work conditions and organizational factors that may influence their psychological health.

Among the most influential contemporary perspectives, the Job Demands–Resources (JD-R) model proposed by Demerouti et al. (14) and further developed by Bakker and Demerouti (15) provides a comprehensive framework for explaining burnout across occupational settings. According to this model, burnout develops when job demands exceed the resources available to workers over prolonged periods. Job demands refer to physical, psychological, social, or organizational aspects of work that require sustained effort and are associated with physiological and psychological costs. In contrast, job resources include organizational support, leadership quality, autonomy, opportunities for development, and other factors that help employees cope with demands and maintain wellbeing. Extensive empirical evidence has demonstrated that high job demands are associated with emotional exhaustion, whereas job resources exert protective effects by reducing burnout and promoting work engagement (16, 17). Recent developments of the Job Demands–Resources framework have further emphasized the dynamic interaction between job demands and organizational resources in shaping employee wellbeing. Demerouti et al. (18) argue that burnout should be understood as a multidimensional process resulting from the imbalance between occupational demands and the availability of resources that enable workers to cope effectively with stressors. This perspective is particularly relevant in high-risk occupational settings such as mining, where psychosocial demands coexist with organizational conditions that may either exacerbate or mitigate burnout symptoms.

Various studies have examined the relationship between occupational factors and burnout from both traditional and emerging perspectives (19). In the healthcare sector, variables such as fatigue, organizational environment, and workload have been identified as key predictors of emotional exhaustion, highlighting the usefulness of machine learning models in detecting complex patterns associated with the syndrome (20, 21). Complementarily, recent research has shown that supervised machine learning techniques facilitate the identification of non-linear relationships among occupational variables, providing complementary evidence to conventional statistical approaches.

Moreover, advances in artificial intelligence have enabled the integration of multiple variables into more robust predictive models, especially in occupational risk environments (22, 23). Recent studies have applied algorithms such as Random Forest, XGBoost, and neural networks to analyze the relationship between occupational exposure, stress, and burnout, achieving accuracy levels above 85% in predicting occupational risks and psychological exhaustion (24). These approaches have demonstrated their capacity to process large volumes of data and detect non-linear interactions between variables, which is essential in complex environments such as mining. However, despite these advances, multiple studies have identified persistent issues associated with burnout, particularly in high-risk sectors (25–27). For instance, research on industrial and mining workers has shown that exposure to factors such as asbestos dust significantly increases the risk of psychological impairment (OR = 1.65; 95% CI: 1.35–2.02), while high levels of burnout are associated with greater probabilities of mental health deterioration (OR = 2.59; 95% CI: 2.39–2.83; *p* < 0.001) (5). These findings highlight the magnitude of the problem and the need for analytical and preventive strategies that enable timely intervention.

Similarly, machine learning-based studies have reported relevant statistical results in burnout prediction (28–30). In the health domain, models such as Gradient Boosting have achieved accuracies of 75.8% in identifying burnout, while variables such as fatigue and perceived organizational leadership have demonstrated strong predictive power (21). Additionally, recent studies indicate that exposure to physical risks (β = 0.76; *p* < 0.01) and prolonged working hours increase the probability of burnout by up to 40%, evidencing the interaction between organizational and environmental factors in the development of the syndrome (31). Previous studies have consistently reported satisfactory validity and reliability indicators for both instruments. The MBI has demonstrated Cronbach's alpha coefficients generally above 0.70 for its dimensions, supporting its stability and internal consistency across occupational groups. Similarly, the SUSESO/ISTAS21 has shown acceptable psychometric performance in Latin American populations, with evidence of construct validity and reliability across its psychosocial dimensions. These characteristics support their suitability for investigating the relationships between psychosocial work conditions and burnout syndrome.

Despite the growing body of evidence, significant gaps remain in the literature, particularly regarding the application of machine learning approaches in extractive sectors such as mining (32, 33). Most studies have focused on healthcare or educational contexts, whereas high-risk industrial environments have been primarily addressed through descriptive or retrospective approaches. Furthermore, there is limited integration of mining-specific psychosocial variables into machine learning models, which restricts the ability to generate contextualized analytical insights and effective preventive strategies. The dimensions assessed in the present study are closely aligned with the JD-R framework. Psychological demands and double presence can be understood as job demands because they reflect workload pressures and work–family conflict. Conversely, social support and leadership quality, as well as active work and skill development, represent organizational resources that may buffer the negative consequences of demanding work conditions. Compensation reflects the balance between effort and reward, which has also been recognized as a relevant organizational determinant of occupational wellbeing.

In this context, it is essential to develop applied research in mining institutions, particularly in operations located in the Moquegua region of Peru, where extractive activities are characterized by intensive work systems, prolonged shift schedules, and simultaneous exposure to physical, environmental, and psychosocial risks. These conditions create a favorable scenario for the emergence of burnout syndrome, as they combine high job demands with complex organizational dynamics typical of large-scale mining. The implementation of machine learning models in these environments not only enables the identification of critical risk factors but also optimizes decision-making in occupational health, improves working conditions, and reduces the incidence of adverse events (34, 35). In this sense, mining in Moquegua, as part of a strategic sector for the Peruvian economy, requires innovative approaches that integrate artificial intelligence and data analysis to strengthen safety, prevent psychosocial risks, and enhance workers' overall wellbeing in highly complex operational contexts.

Consequently, the present study seeks to answer the following research question: What psychosocial risk factors are associated with burnout syndrome among mining workers in Moquegua, Peru, and how effectively can machine learning models identify these relationships? In line with this, the general objective is to analyze the association between psychosocial risk factors and burnout syndrome among mining workers in Moquegua, Peru, using machine learning techniques to identify relevant patterns and relationships that may support occupational health decision-making.

## 2 Materials and methods

The study was conducted under a quantitative, cross-sectional, and analytical approach aimed at examining the associations between psychosocial risk factors and burnout syndrome among mining workers. A non-experimental design was adopted because the variables were observed in their natural work environment without manipulation or intervention by the researchers. Given the cross-sectional nature of the study, all variables were measured at a single point in time; therefore, the analyses do not allow temporal prediction or causal inference. Instead, machine learning techniques were employed as exploratory and analytical tools to identify statistical patterns and classify burnout levels according to workers' psychosocial risk profiles. Consequently, the findings should be interpreted as evidence of concurrent associations rather than as demonstrations of prospective predictive capacity.

For conceptual clarity, it is important to distinguish between psychosocial factors, psychosocial risk factors, and psychosocial risks. Psychosocial factors refer to characteristics of work organization, management practices, social relationships, and working conditions that may influence workers' wellbeing. Psychosocial risk factors correspond to those psychosocial conditions that have the potential to generate adverse physical or psychological outcomes when exposure is prolonged or excessive. Psychosocial risks represent the probability that such adverse outcomes may occur as a consequence of exposure to psychosocial risk factors. In the present study, the SUSESO/ISTAS21 questionnaire was used to assess psychosocial risk factors, whereas burnout syndrome was considered the occupational health outcome associated with these conditions.

The study population consisted of all workers from a mining unit located in the Moquegua region, Peru, during the study period. Due to full accessibility to the population and its manageable size, a census sampling method was employed, ensuring the inclusion of all eligible individuals. Consequently, the final sample comprised 65 workers, representing 100% of the accessible population. Inclusion criteria considered active workers with at least 3 months of experience in the mining operation. Participants with incomplete responses or those who did not provide informed consent were excluded. This sampling strategy reduced selection bias and improved the internal validity of the study. However, it is acknowledged that the sample size may limit the generalizability of the results. Despite this, the census approach ensured comprehensive representation of the evaluated context. The decision to include the entire accessible population was based on the relatively small size of the mining workforce and the objective of maximizing representativeness within the evaluated operational context. Because all eligible workers were invited to participate, the census approach minimized sampling error and reduced the risk of selection bias, providing a more accurate characterization of psychosocial conditions and burnout within the mining unit.

Table 1 presents the sociodemographic and occupational characteristics of the analyzed population, providing a detailed description of the main variables contextualizing the study. The distribution of participants by sex, age, marital status, employment status, and years of service is observed, allowing the identification of relevant patterns within the sample. This information is essential to understand the structural and organizational conditions in which mining workers operate, as well as to properly interpret the results associated with burnout syndrome. Furthermore, the characterization of the sample contributes to establishing the internal validity of the study and delimiting the scope of the findings, particularly in relation to sociodemographic factors that may influence exposure to psychosocial risks.

**Table 1:** Sociodemographic and occupational characteristics of mining workers.

Variable	Category	Frequency	Percentage
Sex	Male	40	61.54
	Female	25	38.46
Age group	< 29 years	15	23.08
	30–45 years	27	41.54
	>45 years	23	35.38
Marital status	Single	35	53.85
	Married	22	33.85
	Divorced	8	12.31
Employment status	Permanent	24	36.92
	Contracted	41	63.08
Years of work	≤ 5 years	36	55.38
	>5 years	29	44.62
Total		65	100.00

Own elaboration based on data collected from mining workers in Moquegua, Peru, during the first quarter of 2026.

Frequencies and percentages correspond to the total sample (n = 65).

Table 1 shows that the sample is pre-dominantly composed of male workers (61.54%), with a higher concentration in the 30–45 age group (41.54%), reflecting a pre-dominantly adult workforce with experience in the mining sector. Additionally, there is a pre-dominance of single workers (53.85%) and non-permanent employment contracts (63.08%), which may be associated with conditions of labor instability. Regarding tenure, more than half of the workers have 5 years or less of experience (55.38%), suggesting a workforce in a consolidation phase. These characteristics indicate a work environment with high physical demands, variability in experience, and potential psychosocial vulnerability factors, which are relevant for understanding burnout syndrome in the mining context. Participants performed operational and technical activities directly related to mining processes, including extraction support, equipment operation, maintenance activities, and field supervision. All workers were exposed to similar organizational conditions, rotating schedules, and occupational demands characteristic of mining operations. Therefore, although specific tasks varied according to job position, participants shared comparable exposure to psychosocial and organizational conditions relevant to the objectives of the study.

Data collection was carried out using standardized and validated instruments widely used in occupational health research. Psychosocial risk factors were assessed using the SUSESO/ISTAS21 short version questionnaire, composed of 20 items distributed across five dimensions: psychological demands (five items), active work and skill development (five items), social support and leadership quality (five items), compensation (three items), and double presence (two items). Burnout syndrome was measured using the Maslach Burnout Inventory (MBI), consisting of 22 items grouped into three dimensions: emotional exhaustion (nine items), depersonalization (five items), and personal accomplishment (eight items). These instruments were selected because they allow a comprehensive evaluation of psychosocial working conditions and burnout syndrome, facilitating the analysis of the relationships between occupational demands, organizational resources, and workers' psychological wellbeing.

Instrument reliability was evaluated using Cronbach's alpha coefficient, widely accepted as an indicator of internal consistency in quantitative studies. The SUSESO/ISTAS21 questionnaire demonstrated acceptable reliability (α = 0.747), indicating adequate coherence among its items. The Maslach Burnout Inventory showed high internal consistency (α = 0.88), confirming its robustness in measuring burnout syndrome. Dimension-level analysis showed reliability values ranging from moderate to high levels, supporting the stability of each construct. Construct validity was ensured through alignment between the instruments and established theoretical models on psychosocial risks and burnout, strengthening the credibility and accuracy of the measurements. Table 2 presents the internal consistency analysis results of the instruments used to measure psychosocial risks and burnout syndrome through Cronbach's alpha coefficient.

**Table 2:** Internal consistency of psychosocial risk and burnout measurement instruments.

Dimensions and subdimensions	Cronbach's alpha	Number of items
Risk factors	0.866	20
Psychological demands	0.711	5
Active work and skill development	0.645	5
Social support and leadership quality	0.625	5
Compensation	0.586	3
Double presence	0.757	2
Burnout syndrome	**0.666**	**22**
Emotional exhaustion	0.953	9
Depersonalization	0.622	5
Personal accomplishment	0.897	8
Total	0.847	42

Own elaboration based on the reliability analysis of the applied instruments.

Cronbach's alpha values indicate the internal consistency of each dimension and subdimension.

Values ≥ 0.70 are considered acceptable, while lower values suggest moderate reliability.

Total alpha (α = 0.847) reflects high overall internal consistency of the instrument (n = 65). Bold values indicate the overall Cronbach's alpha coefficients and the total number of items for the main scales: psychosocial risk factors, burnout syndrome, and the complete instrument. Non-bold values correspond to the Cronbach's alpha coefficients and number of items for each subdimension.

As shown in Table 2, the overall reliability of the instruments was satisfactory (α = 0.847), supporting the internal consistency of the measurement process. However, some subdimensions presented Cronbach's alpha coefficients below the conventional threshold of 0.70, particularly compensation (α = 0.586), social support and leadership quality (α = 0.625), and burnout syndrome (α = 0.666). These values may reflect moderate internal consistency, potential heterogeneity among items, or the multidimensional nature of the assessed constructs. In occupational health research, lower reliability coefficients may also be influenced by the limited number of items included in certain dimensions, especially compensation and double presence. Therefore, the findings associated with these subdimensions should be interpreted with caution. Nevertheless, these reliability levels are comparable to those reported in previous occupational studies employing the SUSESO/ISTAS21 and MBI instruments, supporting their analytical utility while highlighting the need for further psychometric refinement in future research.

**Figure 1:**
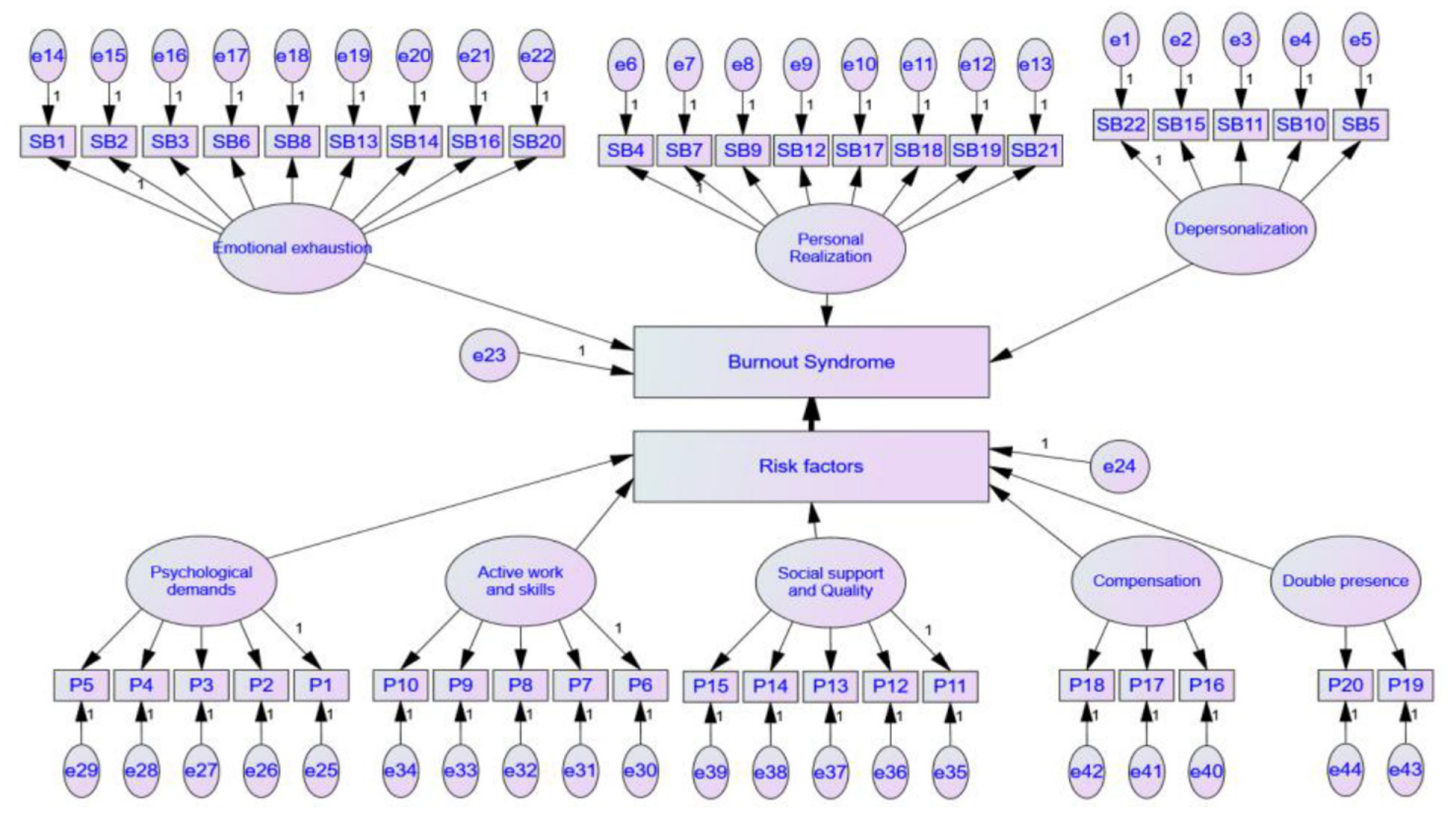
Structural model for measuring psychosocial risks and burnout syndrome. The figure represents the structural measurement model linking psychosocial risk factors and burnout syndrome. Latent variables are operationalized through their respective observed indicators. The model is based on the theoretical framework of Maslach for burnout and the SUSESO/ISTAS21 dimensions for psychosocial risks. Data correspond to mining workers in Moquegua, Peru (*n* = 65).

Figure 1 illustrates the conceptual structure of the instrument, showing the relationships between latent variables and observable indicators. Burnout is represented as a multidimensional construct composed of emotional exhaustion, depersonalization, and personal accomplishment, consistent with Maslach's theoretical model. Psychosocial work conditions were conceptually organized according to the Job Demands–Resources (JD-R) framework. Psychological demands and double presence were considered job demands because they represent sources of occupational strain and work–family conflict. In contrast, active work and skill development, as well as social support and leadership quality, were conceptualized as organizational resources that may buffer the negative effects of job demands and contribute to worker wellbeing. Compensation was considered a contextual organizational factor reflecting the balance between effort and reward. This distinction provides a more coherent interpretation of the relationships between psychosocial conditions and burnout syndrome. This configuration supports the identification of potential relationships among psychosocial risk factors and burnout dimensions, essential for developing machine learning classification models aimed at identifying burnout-related patterns.

The structure presented in Figure 1 demonstrates a coherent theoretical model that articulates psychosocial risk factors with burnout syndrome through hierarchical relationships between latent variables and their observable indicators. Burnout is configured as a multidimensional construct, composed of emotional exhaustion, depersonalization, and personal accomplishment, which aligns with the theoretical foundations of the Maslach model. In turn, psychosocial risk factors are organized into dimensions that reflect both job demands and organizational resources, revealing a complex interaction between working conditions and psychological wellbeing. Consistent with the JD-R model, the dimensions included in the study do not represent homogeneous risk factors. Rather, they comprise both job demands and organizational resources that interact dynamically in the development of burnout syndrome. While psychological demands and double presence increase occupational strain, social support, leadership quality, and opportunities for skill development may function as protective resources that mitigate the adverse effects of demanding work conditions. Consequently, burnout should be understood as the result of the balance between demands and available resources within the organizational environment. This configuration suggests that burnout does not emerge in isolation, but rather as a result of the accumulation and interaction of multiple structural and psychosocial factors. From an analytical perspective, the model enables the identification of potential pathways of influence among variables, which is essential for the development of machine learning analytical models.

Data collection was carried out through an online survey administered via the Google Forms platform, facilitating access and participation among workers. Before starting the questionnaire, participants were presented with a detailed informed consent form explaining the objectives of the study, the voluntary nature of participation, and confidentiality guarantees. Only participants who agreed to the consent were allowed to proceed with the survey. This procedure ensured compliance with ethical principles in human research. Additionally, the study was conducted in accordance with current institutional regulations and received the corresponding authorization. The collected data were anonymized and used exclusively for scientific purposes, ensuring respect for participants' rights and the integrity of the research process.

The collected data were initially exported to Microsoft Excel for coding, cleaning, and verification to ensure data quality. Subsequently, the dataset was processed using SPSS version 26 and complementary tools in Python environments. The pre-processing phase included the identification and treatment of missing values, detection of outliers, and normalization of variables when necessary. Relevant variables were selected based on theoretical and statistical criteria, optimizing the input structure for analytical models. Psychosocial risk dimensions were considered independent variables, while burnout dimensions and the overall score were defined as dependent variables. This procedure improved the quality of the analysis and enhanced the analytical performance of the models used.

A descriptive statistical analysis was conducted to characterize the sample, including measures of central tendency, dispersion, and frequency distribution. Data normality was assessed using the Shapiro–Wilk test to determine the appropriate inferential techniques. Depending on the results, Pearson or Spearman correlation coefficients were used to analyze the relationship between psychosocial risk factors and burnout dimensions. Additionally, multiple linear regression models were applied to identify psychosocial factors significantly associated with burnout syndrome. The level of statistical significance was set at *p* < 0.05. This strategy allowed for the analysis of both the magnitude and direction of associations between variables. The combined use of descriptive and inferential analysis contributed to a comprehensive understanding of the data.

Considering the relatively small population size (*N* = 65), the application of machine learning techniques should be interpreted within an exploratory analytical framework rather than as an attempt to develop highly generalizable predictive models. The objective of incorporating machine learning was not to establish definitive predictive performance but to examine the relative contribution of psychosocial risk factors and identify potential patterns associated with burnout syndrome. Because the study adopted a census approach that included the entire accessible population, the available sample represented the complete operational context under investigation. To mitigate the risk of overfitting and improve model stability, cross-validation procedures and multiple performance metrics were applied. Nevertheless, the findings should be interpreted with caution, as larger samples are generally required to obtain more stable and externally generalizable machine learning estimates.

For the machine learning analysis, the dataset was randomly divided into training (80%) and testing (20%) subsets using a fixed random seed (random_state = 42) to ensure reproducibility. Prior to model training, data quality procedures were conducted, including verification of missing values, detection of outliers, and assessment of variable distributions. No missing values were identified; therefore, imputation procedures were not required. Potential outliers were examined using boxplots and standardized *z*-scores and were retained when considered representative of the target population. Continuous variables were standardized using *z*-score normalization when required by the algorithm. This procedure was particularly applied to the Support Vector Machine (SVM) model because its performance is sensitive to variable scaling. Random Forest and Gradient Boosting models were trained using the original variable scales because tree-based algorithms are less affected by differences in measurement units.

Feature engineering procedures were intentionally limited due to the relatively small sample size. The original psychosocial dimensions obtained from the SUSESO/ISTAS21 questionnaire were used as predictor variables, while the overall burnout score derived from the Maslach Burnout Inventory was used as the outcome variable. No synthetic variables or dimensionality reduction techniques were applied in order to preserve interpretability. Model optimization was performed through Grid Search combined with *k*-fold cross-validation. A stratified 5-fold cross-validation procedure was employed within the training dataset to reduce overfitting and improve the stability of performance estimates. For Random Forest, the number of trees and maximum tree depth were optimized. For Support Vector Machine, kernel type and regularization parameter (C) were evaluated. For Gradient Boosting, the number of estimators, learning rate, and maximum depth were adjusted. The final hyperparameter configuration was selected according to the lowest prediction error observed during cross-validation. Model performance was subsequently evaluated on the independent testing dataset using mean squared error (MSE), root mean squared error (RMSE), mean absolute error (MAE), mean absolute percentage error (MAPE), coefficient of determination (*R*^2^), and coefficient of variation of RMSE (CVRMSE). This procedure provided complementary evidence regarding the relative contribution of psychosocial risk factors associated with burnout syndrome while minimizing the influence of model overfitting.

To reduce the risk of overfitting, hyperparameter optimization was conducted exclusively within the training dataset. The independent testing dataset remained completely isolated throughout model development and was used solely for the final evaluation of model performance. Consequently, no information from the testing subset was incorporated during model training, pre-processing, feature selection, or hyperparameter optimization. Hyperparameter tuning was performed using Grid Search combined with stratified 5-fold cross-validation within the training dataset. During each iteration, four fold were used for model training and one fold for validation, ensuring that model selection was based exclusively on validation performance. This procedure contributed to reducing model variance and improving the stability of performance estimates.

## 3 Results and discussion

This section presents and analyzes the main findings derived from the study, with the aim of evidencing the relationships between psychosocial risk factors and burnout syndrome in the mining context. The results are systematically presented through tables and figures, allowing the identification of relevant patterns and significant trends within the analyzed sample. Subsequently, a critical discussion of the findings is conducted by contrasting them with recent scientific literature, enabling the interpretation of their scope and relevance in the field of occupational health. Additionally, machine learning analyses were used as complementary exploratory tools to identify patterns associated with burnout syndrome, whereas regression models were employed to explain the associations between psychosocial risk factors and burnout dimensions.

**Figure 2:**
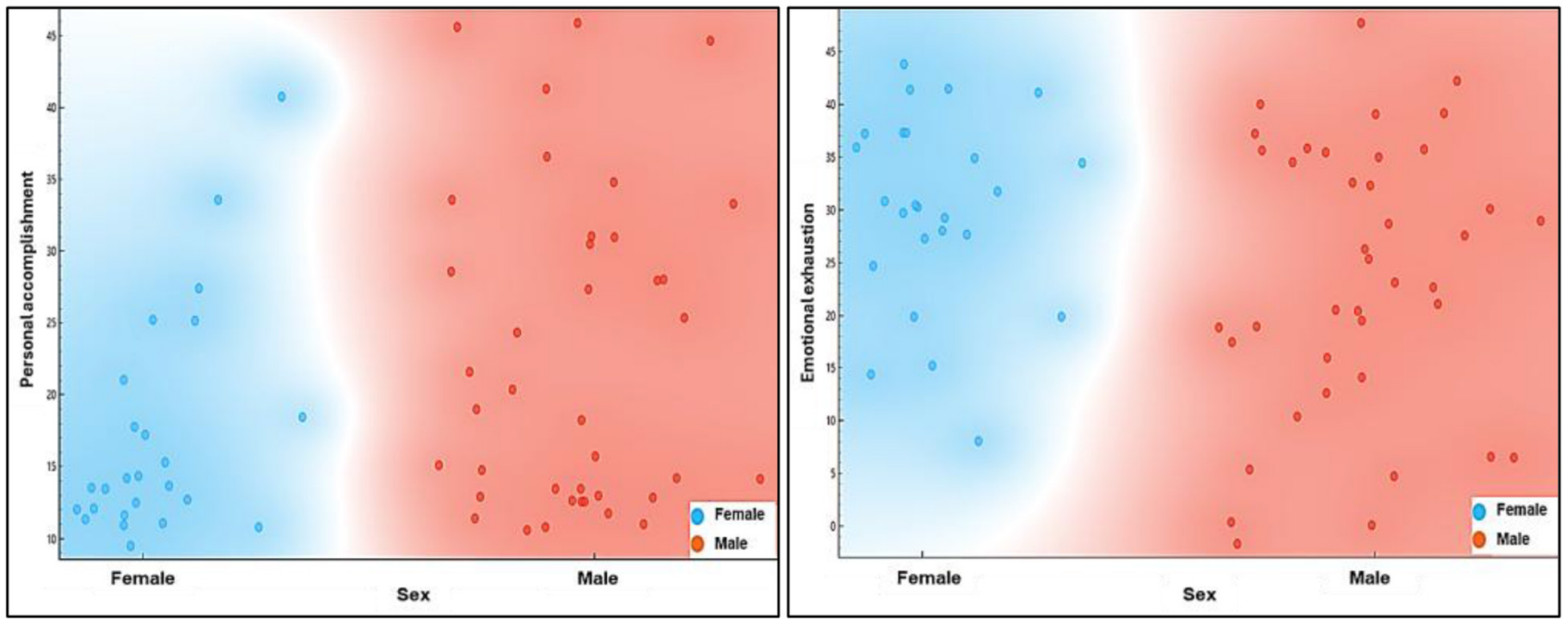
Distribution of personal accomplishment and emotional exhaustion dimensions by sex among mining workers. The figure illustrates the distribution and dispersion of personal accomplishment and emotional exhaustion by sex, highlighting variability patterns and potential differences between groups.

Figure 2 shows the distribution of values corresponding to the dimensions of personal accomplishment and emotional exhaustion according to sex, allowing visualization of data dispersion and potential differential patterns between men and women in the mining context. The graphical representation facilitates the identification of clusters and trends in each variable, as well as the variability within each group. This type of analysis is relevant for complementing inferential results, as it enables a more detailed interpretation of the behavior of burnout dimensions. Furthermore, the visualization contributes to exploring potential structural differences in the manifestation of the syndrome by sex, strengthening the understanding of the phenomenon from both descriptive and analytical perspectives.

Figure 2 shows a higher concentration of male workers at elevated levels of emotional exhaustion and a lower perception of personal accomplishment, whereas the female group presents a more heterogeneous distribution with a tendency toward relatively more favorable levels of achievement. This pattern suggests that sex influences how occupational strain is experienced, which may be associated with task intensity, differential exposure to physical risks, and the organizational structure of mining work. Sun et al. (36) report that burnout is highly prevalent among mining workers and varies significantly according to sociodemographic variables, with higher levels observed in men. This finding supports the notion that the sector's operating conditions characterized by high physical demands and production pressure intensify emotional exhaustion and reduce perceived personal efficacy. Moreover, Garrido-Hermosilla et al. (37) define burnout as a syndrome composed of emotional exhaustion, depersonalization, and low personal accomplishment, which supports the interpretation that the two dimensions analyzed are interrelated and respond to the same process of progressive strain.

Regarding explanatory factors, the observed distribution reflects the influence of psychosocial, organizational, and contextual variables. Imron and Abadi (38) indicate that occupational stress, effort–reward imbalance, and work–family conflict significantly influence the development of burnout, supporting the argument that the accumulation of demands without adequate compensation increases emotional fatigue and reduces work motivation. This interpretation is reinforced by the findings of Samant et al. (39), who highlight that extreme working conditions, prolonged shifts, and production pressure lead to sustained deterioration of psychological wellbeing. In addition, the quality of workplace relationships and organizational leadership act as moderating factors of emotional exhaustion; Schermuly and Meyer (40) demonstrate that supportive and empowering relationships significantly reduce such strain, whereas leadership styles focused solely on outcomes may exacerbate it. In this line, Niu et al. (41) show that economically driven leadership increases emotional exhaustion and compromises safe behaviors among mining workers, suggesting a direct organizational effect on occupational health. Complementarily, Mashaba and Botha (42) emphasize that unfavorable working conditions and imbalances in participation affect work engagement, helping to explain the decrease in personal accomplishment.

Table 3 presents the results of the Pearson correlation analysis, whose purpose is to assess the association between psychosocial risk factors and the dimensions of burnout syndrome among mining workers. It reports the coefficients of determination (*R*^2^), levels of statistical significance (*p*-value), and sample size, enabling identification of the magnitude and direction of the relationships between the analyzed variables. This analysis is essential to determine which psychosocial factors exert the greatest influence on emotional exhaustion, depersonalization, personal accomplishment, and burnout syndrome as a whole.

**Table 3:** Association between psychosocial risk factors and dimensions of burnout syndrome using Pearson correlation.

Risk factors	Pearson test	Burnout syndrome			
		Emotional exhaustion	Depersonalization	Personal accomplishment	Burnout syndrome
Psychological demands	*R* ^2^	0.411^**^	0.368^**^	−0.025	0.546^**^
	*p*-value	0.001	0.003	0.841	0.000
	*N*	65	65	65	65
Active work and skill development	*R* ^2^	0.084	0.173	0.233	0.360^**^
	*p*-value	0.506	0.168	0.062	0.003
	*N*	65	65	65	65
Social support and leadership quality	*R* ^2^	0.452^**^	0.464^**^	−0.029	0.620^**^
	*p*-value	0.000	0.000	0.822	0.000
	*N*	65	65	65	65
Compensation	*R* ^2^	0.323^**^	0.286^*^	−0.196	0.268^*^
	*p*-value	0.009	0.021	0.117	0.031
	*N*	65	65	65	65
Double presence	*R* ^2^	0.368^**^	0.520^**^	0.087	0.652^**^
	*p*-value	0.003	0.000	0.490	0.000
	*N*	65	65	65	65

Own elaboration based on Pearson correlation analysis.

R^2^ values indicate the strength and direction of the association between psychosocial risk factors and burnout dimensions.

Statistical significance levels are denoted as ^*^p < 0.05 and ^**^p < 0.01.

Table 3 shows statistically significant associations between several psychosocial risk factors and the dimensions of burnout syndrome, with moderate positive correlations between psychological demands, social support, double presence, and emotional exhaustion, depersonalization, and the overall burnout index. Notably, higher scores in this dimension reflect deficiencies in organizational support and leadership quality rather than the presence of supportive organizational resources, indicating that both organizational conditions and the interaction between work and family demands create scenarios of high psychosocial vulnerability. Although social support and leadership quality were included within the psychosocial assessment framework, these dimensions are conceptually different from traditional risk factors because they represent organizational resources. Their strong association with burnout suggests that the absence or inadequacy of these resources may increase workers' vulnerability to emotional exhaustion and depersonalization. Therefore, the findings should not be interpreted as indicating that social support itself constitutes a risk, but rather that insufficient support and ineffective leadership are associated with higher burnout levels. Consistent with these findings, Ilić et al. (43) report that psychosocial risks derived from high job demands, low autonomy, and limited social support are directly associated with higher levels of burnout. This perspective supports the argument that the accumulation of job demands without adequate regulatory and support mechanisms increases the likelihood of emotional exhaustion, progressively deteriorating occupational health and workers' coping capacity.

On the other hand, personal accomplishment does not show significant associations with most of the analyzed factors, suggesting that this dimension responds to more complex variables linked to individual resources and protective organizational conditions. In this regard, Moreira et al. (44) identify low job control, dissatisfaction with management, and limited autonomy as critical factors in the development of burnout, reinforcing the relevance of leadership and organizational structure highlighted in the results. Likewise, Guerrero-Barona et al. (45) emphasize that job satisfaction, social support, and emotional intelligence act as protective resources that mitigate the effects of job demands, allowing the weak relationship with personal accomplishment to be interpreted as the result of these moderating factors.

Complementarily, Zivković et al. (46) argue that psychosocial risks explain a significant proportion of the variance in occupational stress, evidencing their structural impact on occupational health. Furthermore, other studies included in the documentary corpus, particularly those focused on analyzing working conditions, stress, and professional burnout in complex organizational contexts, consistently indicate that the interaction between job demands, available resources, and environmental conditions constitutes the core explanatory framework of burnout. This reinforces the need for comprehensive interventions focused on psychosocial risk management and the strengthening of workplace support systems.

Table 4 presents the results of the multiple linear regression model, whose objective is to identify the association between psychosocial risk factors and burnout syndrome. It reports unstandardized and standardized coefficients, *t*-values, levels of statistical significance, and 95% confidence intervals, allowing evaluation of the magnitude, direction, and precision of the estimated relationships. This analysis is essential to determine which dimensions of psychosocial risks significantly contribute to explaining burnout, as well as to validate the hypotheses proposed in the study.

**Table 4:** Linear regression model of psychosocial risk factors associated with burnout syndrome in mining workers.

Predictors	Unstandardized coefficients		Standardized coefficients	*t*	*p*-value	95.0% confidence interval for *B*		Hypothesis threshold
	*B*	Std. error	Beta			Lower CI	Upper CI	
Psychological demands	1.818	0.351	0.546	5.174	0.000	1.116	2.520	Accepted
Active work and skill development	1.109	0.362	0.360	3.061	0.003	0.385	1.832	Accepted
Social support and leadership quality	1.979	0.316	0.620	6.268	0.000	1.348	24610	Accepted
Compensation	1.209	0.547	0.268	2.211	0.031	0.116	2.302	Accepted
Double presence	3.938	0.577	0.652	6.821	0.000	2.784	5.092	Accepted
Psychosocial risks (overall)	0.614	0.096	0.628	6.407	0.000	0.423	0.806	Accepted

Own elaboration based on multiple linear regression analysis. *B* represents unstandardized coefficients, while Beta indicates standardized coefficients.

Statistical significance was set at *p* < 0.05.

Confidence intervals (95% CI) reflect the precision of the estimates.

Table 4 shows that all psychosocial risk factors included in the model exhibit significant associations with burnout syndrome, with high standardized coefficients, particularly for double presence, social support and leadership quality, and overall psychosocial risks. These results suggest that occupational strain does not depend solely on direct job demands but rather on a complex network of organizational and personal conditions that interact simultaneously. In this regard, Zhao et al. (47) demonstrate that occupational stress, burnout, and depression are significant predictors of health problems among mining workers, implying that the observed impact in the model coefficients reflects a cumulative process of exposure to adverse working conditions that progressively deteriorate mental health. Likewise, Mościcka-Teske et al. (48) indicate that high levels of psychosocial risk are associated with lower job satisfaction and increased stress, supporting the inference that the significance of variables such as psychological demands and leadership reflects work environments with limited emotional regulation and organizational support capacity. Complementarily, Li et al. (49) show that occupational stress increases the risk of physical and psychological impairments among miners, reinforcing the interpretation that the high coefficients are not isolated events but indicators of a structurally demanding work system.

From the perspective of the Job Demands–Resources model, these findings reinforce the notion that burnout is not exclusively determined by workload but also by the availability of organizational resources. Demerouti et al. (18) indicates that leadership quality, social support, and opportunities for development may buffer the effects of occupational strain, which is consistent with the significant associations identified in the present study. On the other hand, the relevance of double presence and compensation highlights the role of work–life conflict and effort–reward imbalance in the configuration of burnout. In this framework, Nyaaba et al. (50) report that work-related stress is associated with anxiety and depression, suggesting that double presence intensifies these effects by generating simultaneous overload that is difficult to manage. Similarly, Lestari et al. (51) identify that psychosocial factors such as organizational trust directly influence psychological wellbeing, indicating that the weight of leadership in the model reflects its function as either a protective resource or a risk factor depending on its quality. In the same line, Deng et al. (52) argue that work–family conflict and effort–reward imbalance increase burnout, explaining the significance of compensation in the estimated model. Finally, Lu et al. (53) highlights that burnout and occupational stress are key variables in predicting mental health problems among industrial workers, confirming that the results obtained correspond to widely documented patterns in demanding work environments.

Table 5 presents the results of the multiple linear regression analysis aimed at evaluating the association between psychosocial risk factors and emotional exhaustion on the emotional exhaustion dimension among mining workers. It reports unstandardized and standardized coefficients, *t*-values, levels of statistical significance, and 95% confidence intervals, allowing analysis of the magnitude, direction, and precision of the relationships between variables. This analysis is essential to identify which psychosocial factors significantly contribute to emotional exhaustion, considered one of the core dimensions of burnout syndrome.

**Table 5:** Linear regression model of psychosocial risk factors associated with emotional exhaustion in mining workers.

Predictors	Unstandardized coefficients	Std. error	Standardized coefficients	*t*	*p*-value	95.0% confidence interval for *B*	Upper CI	Hypothesis threshold
	*B*		Beta			Lower CI		
Psychological demands	1.484	0.415	0.411	3.580	0.001	0.656	2.313	Accepted
Active work and skill development	0.281	0.419	0.084	0.669	0.506	−0.557	1.119	Not Accepted
Social support and leadership quality	1.565	0.389	0.452	4.021	0.000	0.787	2.343	Accepted
Compensation	1.577	0.583	0.323	2.708	0.009	0.413	2.741	Accepted
Double presence	2.409	0.768	0.368	3.138	0.003	0.875	3.943	Accepted
psychosocial risks (overall)	0.441	0.122	0.415	3.625	0.001	0.198	0.683	Accepted

Own elaboration based on multiple linear regression analysis.

*B* represents unstandardized coefficients, while Beta indicates standardized coefficients.

Statistical significance was set at *p* < 0.05.

Confidence intervals (95% CI) indicate the precision of the estimates.

Table 5 shows that emotional exhaustion is significantly determined by multiple psychosocial risk factors, highlighting social support and leadership quality, overall psychosocial risks, psychological demands, and double presence as relevant predictors, while active work and skill development does not reach statistical significance. This pattern suggests that emotional exhaustion is not solely a response to objective workload but rather to the interaction between emotional demands, organizational resources, and contextual working conditions. In this regard, Yong et al. (54) indicate that mining workers experience high levels of occupational strain associated with cumulative labor factors, supporting the interpretation that the significance of psychological demands and psychosocial risks reflects work environments characterized by prolonged exposure to adverse conditions affecting both physical and mental health. These findings also align with the JD-R framework, according to which emotional exhaustion represents the primary energetic consequence of prolonged exposure to excessive job demands. As suggested by Demerouti et al. (18), emotional exhaustion tends to intensify when organizational resources are insufficient to offset the impact of demanding working conditions.

Furthermore, Aragundi-Moncada et al. (55) emphasize the importance of emotional competencies in high-demand contexts, implying that the absence of adequate socio-emotional resources may intensify emotional exhaustion in demanding work environments. Complementarily, Gao et al. (56) show that health-related factors such as sleep quality significantly influence burnout, suggesting that mining working conditions generate cumulative effects that extend beyond the organizational domain and directly impact psychological wellbeing. On the other hand, the significance of social support and leadership quality confirms the role of organizational resources as protective or moderating factors of emotional exhaustion, while double presence reflects the tension between work and family demands. In this framework, Zhu et al. (57) demonstrate that role demands and psychosocial stress directly influence worker performance and wellbeing, allowing the inference that role overload and ambiguity intensify emotional exhaustion in mining contexts. Likewise, Petersen et al. (58) show that job demands and low organizational cooperation are critical factors in the development of emotional exhaustion, supporting the importance of leadership and social support identified in the model. Similarly, Yu et al. (59) indicate that occupational stress and burnout interact to increase the risk of psychological disorders, suggesting that the combination of significant factors in the model amplifies workers' emotional deterioration. Finally, Chapman et al. (60) highlight that individual factors and coping strategies influence the development of burnout, indicating that emotional exhaustion depends not only on external conditions but also on the individual's capacity to manage stress.

Table 6 presents the results of the multiple linear regression analysis aimed at evaluating the effect of psychosocial risk factors on the depersonalization dimension among mining workers. It reports unstandardized and standardized coefficients, *t*-values, levels of statistical significance, and 95% confidence intervals, allowing examination of the magnitude, direction, and precision of the associations between the analyzed variables. This analysis is essential to identify which psychosocial factors significantly influence depersonalization, understood as one of the core dimensions of burnout syndrome. Furthermore, the results enable the testing of the proposed hypotheses and strengthen the validity of the explanatory model, providing relevant empirical evidence for understanding the effects of working conditions on the psychological strain of mining workers.

**Table 6:** Psychosocial risk factors associated with depersonalization.

Predictors	Unstandardized coefficients	Std. error	Standardized coefficients	*t*	*p*-value	95.0% confidence interval for *B*	Upper CI	Hypothesis threshold
	*B*		Beta			Lower CI		
Psychological demands	0.410	0.131	0.368	3.139	0.003	0.149	0.671	Accepted
Active work and skill development	0.179	0.128	0.173	1.395	0.168	−0.077	0.435	Not Accepted
Social support and leadership quality	0.496	0.119	0.464	4.156	0.000	0.258	0.735	Accepted
Compensation	0.432	0.182	0.286	2.370	0.021	0.068	0.796	Accepted
Double presence	1.052	0.218	0.520	4.828	0.000	0.617	1.488	Accepted
Psychosocial risks (overall)	0.148	0.037	0.451	4.013	0.000	0.074	0.221	Accepted

Own elaboration based on multiple linear regression analysis.

*B* represents unstandardized coefficients, while Beta indicates standardized coefficients.

Statistical significance was set at *p* < 0.05. Confidence intervals (95% CI) indicate the precision of the estimates.

Table 6 shows that depersonalization is configured as a dimension of burnout significantly influenced by psychosocial risk factors, with double presence, social support and leadership quality, overall psychosocial risks, and psychological demands emerging as statistically significant associated factors, while active work and skill development does not show a relevant effect. This pattern indicates that depersonalization does not arise in isolation but rather as a dysfunctional adaptive response to highly demanding work environments with insufficient organizational support. In this regard, Lu et al. (61) demonstrate that prolonged exposure to occupational risks and work-related stress significantly increases the likelihood of burnout among mining workers.

Based on this finding, it can be interpreted that the significance of psychological demands and psychosocial risks reflects working conditions that generate progressive strain, promoting the adoption of emotional distancing as a defense mechanism. Likewise, Fu et al. (62) identify that occupational stress and burnout have direct effects on psychological symptoms, suggesting that depersonalization functions as an intermediate component translating stress into psychological deterioration. Complementarily, Lin et al. (63) highlight the interaction between environmental factors and coping styles in the development of burnout, indicating that the variability observed in depersonalization may also be explained by individual differences in managing work-related stress. This interpretation is consistent with Demerouti et al. (18), who propose that depersonalization may emerge as a coping mechanism when workers experience persistent occupational strain and inadequate organizational support. Consequently, the significant role of psychosocial demands and organizational resources identified in this study reinforces the explanatory value of the JD-R model.

On the other hand, the relevance of social support and leadership quality as predictors reinforces the idea that depersonalization can be modulated by organizational factors, particularly those related to institutional support structures. In this framework, Matulić et al. (64) indicate that psychosocial stressors in the work environment directly affect workers' functional capacity and wellbeing, suggesting that lack of support increases the likelihood of developing interpersonal detachment. Similarly, Alexaki et al. (65) identify depersonalization as one of the core dimensions of burnout associated with organizational and demographic factors, supporting the consistency of the results obtained. Finally, Elsaie et al. (66) show that burnout develops progressively from multiple work-related stressors, leading to consequences such as emotional distancing and reduced professional engagement. From this theoretical and empirical integration, it is argued that depersonalization among mining workers constitutes a complex, multifactorial response highly dependent on the organizational context, requiring interventions aimed at strengthening social support, improving working conditions, and promoting effective coping strategies to mitigate the impact of occupational stress.

Table 7 presents the results of the multiple linear regression analysis aimed at evaluating the effect of psychosocial risk factors on the personal accomplishment dimension among mining workers. It reports unstandardized and standardized coefficients, *t*-values, levels of statistical significance, and 95% confidence intervals, allowing examination of the magnitude, direction, and precision of the relationships between the analyzed variables. This analysis is relevant to determine whether working conditions and psychosocial factors influence perceived personal accomplishment, considered a key dimension of burnout syndrome. Furthermore, the results allow testing of the proposed hypotheses and evaluation of the explanatory capacity of the model, providing empirical evidence to understand the role of organizational factors in the development of occupational wellbeing in the mining context.

**Table 7:** Linear regression model of psychosocial risk factors associated with personal accomplishment.

Predictors	Unstandardized coefficients	Std. error	Standardized coefficients	*t*	*p*-value	95.0% confidence interval for *B*	Upper CI	Hypothesis threshold
	*B*		Beta			Lower CI		
Psychological demands	−0.077	0.380	−0.025	−0.202	0.841	−0.836	0.682	Not accepted
Active work and skill development	0.649	0.342	0.233	1.898	0.062	−0.034	1.332	Not accepted
Social support and leadership quality	−0.082	0.364	−0.029	−0.226	0.822	−0.810	0.645	Not accepted
Compensation	−0.800	0.504	−0.196	−1.587	0.117	−1.808	0.207	Not accepted
Double presence	0.477	0.687	0.087	0.694	0.490	−0.896	1.850	Not accepted
Psychosocial risks (overall)	0.026	0.112	0.029	0.231	0.818	−0.197	0.249	Not accepted

Own elaboration based on multiple linear regression analysis.

*B* represents unstandardized coefficients, while Beta indicates standardized coefficients. Statistical significance was set at *p* < 0.05.

Confidence intervals (95% CI) indicate the precision of the estimates.

Table 7 shows that psychosocial risk factors do not exhibit significant associations for personal accomplishment among mining workers, as none of the coefficients reach statistical significance. This result suggests that, unlike other dimensions of burnout syndrome, personal accomplishment responds to more complex dynamics that depend not only on adverse working conditions but also on individual resources, subjective perceptions, and contextual variables. In this regard, Amponsah-Tawiah et al. (67) indicate that psychosocial risks directly influence overall wellbeing, although their effects may vary depending on demographic factors and individual perceptions of the work environment.

Based on this, the absence of significant association in this dimension may be explained by workers' ability to maintain levels of self-efficacy or a sense of achievement even in demanding contexts. Complementarily, Liu et al. (68) highlight that internal psychological factors, such as psychological capital, have differentiated effects on work behaviors and outcomes, suggesting that personal accomplishment may be more strongly linked to internal resources than to external conditions. Likewise, Okello et al. (69) show that job demands influence physical health and performance but do not necessarily directly affect perceived personal achievement, reinforcing the multidimensional nature of this variable.

On the other hand, the lack of significance of variables such as social support, compensation, or double presence suggests that personal accomplishment does not automatically deteriorate under unfavorable working conditions but may remain stable through coping and adaptation mechanisms. In this sense, James et al. (70) identify that psychological distress among mining workers is associated with specific job-related factors, although not all of them affect all dimensions of wellbeing equally. This finding supports the notion that personal accomplishment may function as a more resilient component within burnout.

Similarly, Considine et al. (71) emphasize that individual, social, and organizational characteristics interact differently in mental health, implying that certain constructs, such as personal satisfaction, may depend on specific combinations of factors. Finally, Korneeva et al. (72) highlight that psychosocial factors can both negatively affect and enhance wellbeing and work capacity depending on their configuration. Based on this evidence, it is argued that personal accomplishment among mining workers constitutes a relatively stable dimension, modulated by internal resources, which underscores the need to design interventions that strengthen psychological capital and the sense of achievement beyond merely reducing psychosocial risks.

Table 8 presents the analytical performance obtained for the psychosocial risk factors included in the machine learning model. The reported indicators (MSE, RMSE, MAE, MAPE, *R*^2^, and CVRMSE) allow the evaluation of the relative contribution of each psychosocial dimension to the identification of burnout-related patterns among mining workers. This analysis provides complementary evidence regarding the relevance of different psychosocial factors in explaining burnout syndrome within the analyzed dataset.

**Table 8:** Relative contribution of psychosocial risk factors associated with burnout syndrome.

Model	MSE	RMSE	MAE	MAPE	*R* ^2^	CVRMSE
Psychological demands	81.896	9.050	7.081	0.140	0.298	16.730
Active work and skill development	101.593	10.079	7.902	0.156	0.129	18.634
Social support and leadership quality	71.879	8.478	6.740	0.133	0.384	15.673
Compensation	108.296	10.407	8.363	0.165	0.072	19.238
Double presence	67.129	8.193	6.559	0.131	0.425	15.147
Psychosocial risks (overall)	70.656	8.406	6.574	0.132	0.395	15.540

Own elaboration based on machine learning analysis of psychosocial risk factors associated with burnout syndrome.

MSE, mean squared error; RMSE, root mean squared error; MAE, mean absolute error; MAPE, mean absolute percentage error; R^2^, coefficient of determination; CVRMSE, coefficient of variation of RMSE.

Table 8 indicates that psychosocial factors such as double presence, social support and leadership quality, and overall psychosocial risks exhibit the strongest associations with burnout-related patterns, as reflected by lower error values and higher coefficients of determination. In contrast, compensation and active work and skill development show weaker explanatory contributions. These findings suggest that organizational support, work–family conflict, and psychosocial working conditions play a central role in the manifestation of burnout among mining workers. Consistent with these findings, Tian et al. (73) demonstrate that emotional regulation strategies and working conditions significantly influence work fatigue and risk decision-making. This supports the argument that model accuracy depends not only on structural variables but also on psychological factors that mediate workers' responses to demanding environments. Likewise, Li et al. (74) highlight that the incorporation of machine learning–based predictive models improves the identification of risk factors among mining workers, supporting the relevance of using multiple indicators to optimize predictive performance. In this sense, the variability observed in error indicators may be explained by the heterogeneity of psychosocial factors and their differential impact on occupational health. It is important to emphasize that the machine learning results should not be interpreted as evidence of prospective prediction. Instead, these models were used to explore patterns within the available data and to assess the relative contribution of psychosocial risk factors associated with burnout syndrome.

The machine learning findings also support the theoretical assumptions of the JD-R framework. Demerouti et al. (18) argue that burnout cannot be explained by isolated occupational factors but rather by the interaction between demands and resources. In this study, dimensions related to work–family conflict, leadership, and organizational support demonstrated the strongest analytical contribution, highlighting the importance of considering both risk and protective factors when examining burnout syndrome. On the other hand, the model's behavior confirms that occupational stress and organizational conditions act as key determinants in the emergence of burnout, although their relative weight varies depending on the dimension analyzed. Yu and Li (75) show that a psychosocial safety climate reduces unsafe behaviors through the mediation of work stress and burnout, suggesting that organizational factors can modulate the intensity of these phenomena.

Similarly, Zhang et al. (76) report that occupational stress is associated with alterations in physical and mental health, reinforcing the need to integrate workload and reward variables into predictive models. Complementarily, An et al. (77) indicate that factors such as stress, anxiety, and working conditions directly influence workers' functional capacity, suggesting that model accuracy is affected by individual variables not always captured in traditional indicators. Finally, Fernandes et al. (78) emphasize that psychosocial risks are determined by work organization, interpersonal relationships, and working conditions, and that their proper modeling requires comprehensive approaches. Based on this evidence, it is argued that the performance of the predictive model is consistent with the literature, although its optimization requires the integration of psychosocial, organizational, and personal variables to capture the complexity of burnout in highly demanding work environments.

A limitation of this study is the relatively small sample size available for machine learning analyses. Although the census approach ensured the inclusion of all accessible workers within the mining unit, the limited number of observations may affect model stability, increase the risk of overfitting, and restrict the generalizability of the findings. Consequently, the machine learning results should be interpreted as exploratory evidence of associations between psychosocial risk factors and burnout rather than as definitive predictive models. Future studies should include larger and more diverse samples from multiple mining operations to improve model robustness and external validity.

## 4 Conclusion

The present study examined the association between psychosocial risk factors and burnout syndrome among mining workers through the application of statistical and machine learning techniques. The findings indicate that psychosocial dimensions such as double presence, social support and leadership quality, psychological demands, and compensation are strongly associated with burnout levels. While machine learning models demonstrated an adequate capacity to classify burnout patterns within the analyzed dataset, the cross-sectional design prevents interpreting these results as evidence of prospective prediction. Consequently, the findings should be understood as identifying concurrent relationships between psychosocial risks and burnout rather than forecasting future burnout occurrence. Furthermore, the machine learning findings should be interpreted as exploratory analytical evidence derived from a census-based sample rather than as highly generalizable predictive models, highlighting the need for future studies with larger populations and longitudinal designs.

Regarding the main findings, it was determined that psychosocial risk factors exert a significant influence on the development of burnout, with double presence, social support, and leadership quality emerging as the most strongly associated factors in both statistical models and machine learning algorithms. Similarly, psychological demands and compensation showed relevant effects, confirming that occupational strain results from a multifactorial interaction among job demands, organizational resources, and personal conditions. Additionally, the dimensions of emotional exhaustion and depersonalization were found to be highly sensitive to these factors, whereas personal accomplishment did not show a significant relationship, suggesting a greater dependence on individual variables and internal resources.

The findings also highlight the importance of distinguishing between psychosocial demands and organizational resources when analyzing burnout syndrome. While psychological demands and double presence were associated with increased occupational strain, dimensions such as social support, leadership quality, and opportunities for skill development should be interpreted as protective organizational resources. This distinction contributes to a more comprehensive understanding of burnout and aligns the findings with the Job Demands–Resources theoretical framework. Overall, the results are consistent with the Job Demands–Resources framework and contribute to the growing body of evidence indicating that burnout emerges from the interaction between excessive job demands and insufficient organizational resources. By extending this perspective to the mining sector, the study provides empirical evidence from a high-risk occupational context that has received comparatively limited attention in the burnout literature.

However, the study presents certain limitations that should be considered. First, the relatively small sample size and the census approach limited to a single mining unit restrict the generalizability of the findings to other industrial or geographical contexts. Second, the cross-sectional design does not allow for the establishment of definitive causal relationships, limiting the analysis to associations and associations observed at a specific point in time. Furthermore, reliance on self-reported instruments may introduce perception bias or social desirability bias in the responses.

Based on the above, future research directions are proposed to expand the sample size and diversity by incorporating multiple mining units and work contexts, thereby strengthening the external validity of the findings. Likewise, the development of longitudinal studies is recommended to analyze the evolution of burnout over time and to validate the stability of machine learning classification models. Finally, it is advisable to integrate additional variables, such as biological factors, advanced organizational variables, and real-time metrics, as well as to explore hybrid models and more advanced artificial intelligence techniques, in order to optimize predictive accuracy and contribute to the design of more effective preventive strategies in occupational health.

## Data Availability

The original contributions presented in the study are included in the article/supplementary material, further inquiries can be directed to the corresponding author.
